# Effects of Yttria Content and Margin Design on the Fracture Resistance of Monolithic Zirconia Crowns

**DOI:** 10.3390/jfb17050219

**Published:** 2026-05-02

**Authors:** Beyza Güney, Elif Yılmaz Biçer, Dilan Gizem Doğan, Merve Bankoğlu Güngör

**Affiliations:** Department of Prosthodontics, Faculty of Dentistry, Gazi University, 06490 Ankara, Türkiye; beyzabayat@gazi.edu.tr (B.G.); elifyilmaz1@gazi.edu.tr (E.Y.B.); gizemdogan@gazi.edu.tr (D.G.D.)

**Keywords:** multilayer zirconia, monolithic crown, yttria concentration, margin design, fracture resistance

## Abstract

Background: Zirconia ceramics are generally used in monolithic restorations, and their microstructural, mechanical, and optical properties continue to improve. Several factors affect the mechanical properties of these restorations; however, the combined effects of yttria content and margin design on the fracture resistance remain unclear. Methods: Sixty monolithic zirconia crowns were fabricated and assigned to six groups (*n* = 10) based on three different yttria contents (strength-gradient multilayer zirconia containing 3 mol% yttria tetragonal zirconia polycrystals in the dentin region and 5 mol% yttria-partially stabilized zirconia in the occlusal region: 3Y-TZP/5Y-PSZ [ZP], 3 mol% yttria tetragonal zirconia polycrystals: 3Y-TZP [HTML], and 4 mol% yttria-partially stabilized zirconia: 4Y-PSZ [STML]), and two different margin designs (chamfer and rounded shoulder). Crowns were adhesively bonded to standardized 3-dimensional-printed resin dies and subjected to thermal and mechanical aging (10,000 thermocycles at 5–55 °C, and 1.2 million mechanical cycles at 50 N, 1.6 Hz). Fracture resistance values were recorded in Newtons, and fracture types were evaluated. Data were analyzed using a two-way analysis of variance (ANOVA), and Bonferroni adjustment was used for multiple comparisons (α = 0.05). Results: A significant interaction between yttria content and margin design was found (*p* = 0.005). In the chamfer margin design groups, ZP (2208.5 ± 501.9 N) and HTML (2069.6 ± 463.3 N) showed significantly higher fracture resistance than STML (1444 ± 303.2 N) (*p* < 0.05). In the rounded shoulder margin design groups, no significant differences were observed among ZP (1662.8 ± 293.8 N), HTML (1940.9 ± 341.6 N), and STML (1795.6 ± 529.6 N) (*p* > 0.05). ZP and HTML showed higher fracture resistance values with the chamfer margin design, while STML showed higher fracture resistance with the rounded shoulder margin design. Conclusions: The fracture resistance of zirconia restorations is influenced by both the margin design and the yttria content. Designing the margin geometry based on the type of zirconia to be used can enhance the mechanical properties of the restorations and support clinical decision-making.

## 1. Introduction

The yttria (Y_2_O_3_) content in zirconia ceramic restorations plays a critical role in determining their phase composition, optical properties, and mechanical behavior. Zirconia occurs in monoclinic, tetragonal, and cubic crystallographic phases. The tetragonal phase is the most mechanically advantageous and can be retained at room temperature by stabilizing it with yttria [1]. As a stabilizer, yttria influences the proportions of the tetragonal and cubic crystal phases in the zirconia structure. 3 mol% yttria-stabilized zirconia (3Y-TZP) is predominantly composed of the tetragonal phase. 3Y-TZP exhibits high mechanical performance, largely attributed to the transformation toughening mechanism. Under mechanical and thermal stresses, metastable tetragonal grains transform into the monoclinic phase, leading to a localized volumetric expansion that generates compressive stresses at the crack tip and limits crack propagation. However, the optical anisotropy of the tetragonal phase and light scattering at grain boundaries result in relatively low translucency, which limits its indication in esthetic applications. Zirconia with a higher yttria content is generally referred to as partially stabilized zirconia (PSZ) because of its greater prevalence of the cubic phase [2]. Increasing the yttria content to 4 or 5 mol% promotes a higher proportion of cubic phase, which is optically isotropic and reduces light scattering, thereby improving translucency. Nevertheless, because the cubic phase does not contribute to transformation toughening, 5Y-PSZ shows reduced mechanical performance and is less suitable for applications requiring high strength. As a compromise between strength and translucency, 4Y-PSZ has been introduced, providing a more balanced combination of mechanical and optical properties. Its mixed microstructure, comprising both tetragonal and cubic phases, enhances translucency while maintaining adequate mechanical integrity [1,3]. Therefore, high yttria content (4–5 mol%) is more appropriate for anterior restorations, where esthetic outcomes are prioritized over mechanical strength. In response to increasing esthetic demands, recently developed multilayer monolithic zirconia materials exhibit a gradual transition in color and translucency, enabling a more natural tooth-like appearance. Initial approaches achieved this shade gradient structure solely through pigmentation, maintaining the same zirconia generation throughout the blank and thus providing uniform strength across the enamel and dentin regions. More recently, advanced multilayer technologies have been introduced that combine different generations of zirconia within a single disc, integrating high-strength 3Y-TZP in the cervical/dentin region to ensure mechanical stability with highly translucent 5Y-TZP in the incisal or occlusal region to enhance esthetics [3,4]. Understanding the balance among yttria content, translucency, and fracture resistance is crucial for selecting the most suitable zirconia material for clinical requirements.

**Figure 1:**
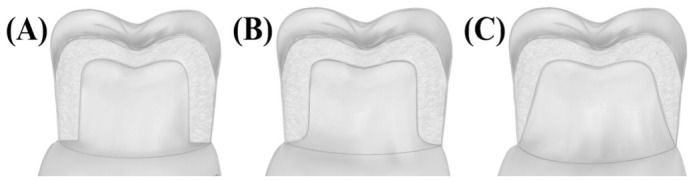
Schematic view of the (**A**) shoulder, (**B**) deep chamfer, and (**C**) feather-edge margin designs.

The mechanical properties of monolithic zirconia restorations are significantly influenced by the design of the finish line or margin preparation. Various margin designs (Figure 1), such as shoulder, deep chamfer, or feather-edge, affect the distribution of stresses within the restoration under functional loading.

It has been shown that a deep chamfer or rounded shoulder design provides better support for zirconia restorations, reducing stress concentration at the margins and enhancing fracture resistance. In contrast, knife-edge or feather-edge designs may result in thinner marginal areas that are more susceptible to chipping or catastrophic failure, especially in high-strength yet brittle ceramics, such as zirconia [5,6]. Additionally, more conservative margins can preserve tooth structure, but must be balanced against the need for sufficient material thickness to maintain strength. As monolithic zirconia does not rely on veneering porcelain, optimizing margin design to leverage its intrinsic strength and minimizing stresses are crucial for long-term clinical success [7]. Mitov et al. [8] evaluated the effect of different preparation designs on the fracture resistance of monolithic zirconia crowns. Three margin designs were tested: shoulderless, 0.4 mm chamfer, and 0.8 mm chamfer. It was indicated that the shoulderless preparation exhibited significantly higher fracture resistance than both chamfer designs, for which no significant difference was observed. Findakly et al. [9] investigated the effect of margin design on the fracture resistance of monolithic zirconia crowns. The authors compared two margin designs (shoulder and feather-edge) in both IPS e.max ZirCAD LT, a conventional 3Y-TZP zirconia, and IPS e.max ZirCAD MT Multi, a multilayer zirconia containing 4Y-PSZ in the cervical region and 5Y-PSZ in the incisal region. Their results demonstrated that crowns with a shoulder margin exhibited significantly higher fracture resistance than those with a feather-edge margin. Moreover, the modes of fracture varied between the groups; shoulder preparations mainly were associated with fractures involving the crown and/or tooth, whereas feather-edge preparations tended to fracture through the central fossa. These findings highlighted that the margin design had an important role in the mechanical properties of zirconia crowns. More recently, Juntavee et al. [7] assessed the effect of margin thickness on the fracture resistance of highly translucent monolithic zirconia crowns. They found that margin thickness significantly influenced both load-bearing capacity and durability, with 1.2 mm chamfer margins exhibiting superior performance compared to 0.8 mm chamfer margins.

Restorative materials are continuously subjected to complex and dynamic mechanical, thermal, and chemical factors in the oral environment that may compromise their long-term clinical performance. Aging and fatigue progressively impair the structural integrity of restorative materials, potentially leading to mechanical degradation and eventual clinical failure [10]. Consequently, the preclinical evaluation of emerging dental materials through well-designed and standardized in vitro studies is essential before their widespread clinical implementation [11]. Despite the increasing clinical use of multilayer monolithic zirconia in oral rehabilitation, current evidence regarding the influence of aging processes on their mechanical behavior remains limited. Therefore, the application of clinically relevant aging protocols, such as thermomechanical dynamic loading, is crucial for a better understanding of their long-term performance. This in vitro aging approach, commonly referred to as mastication simulation, is a well-established methodology for reproducing clinically relevant intraoral conditions [12]. In contrast to conventional single-cycle load-to-failure tests, which may overestimate the mechanical reliability of ceramic materials, this approach provides a more clinically representative assessment by subjecting specimens to cyclic physiological loading and thermally induced stresses. Repeated cyclic loading at stress levels below the ultimate strength can initiate and propagate internal or surface defects, thereby weakening the structural stability of the material. The residual mechanical stability of the aged specimens is subsequently evaluated using a fracture resistance test [2,10].

Previous studies have investigated the mechanical performance of multilayer zirconia materials, particularly fracture resistance [2,3,11,13,14,15,16,17,18]. In addition, the effect of margin design on the fracture resistance of monolithic zirconia restorations has been evaluated [5,7,8,9,19,20,21,22]. However, studies investigating the combined effects of yttria content and margin design on the fracture resistance of multilayer monolithic zirconia systems remain limited. The purpose of the present in vitro study was to evaluate the effects of the yttria contents (3Y-TZP/5Y-PSZ, 3Y-TZP, and 4Y-PSZ) and different margin designs (chamfer and rounded shoulder) on the fracture resistance of monolithic zirconia crowns. Therefore, the null hypothesis was that the yttria content and margin design would not affect the fracture resistance of monolithic zirconia crowns.

## 2. Materials and Methods

### 2.1. Sample Size Calculation and Study Design

**Figure 2:**
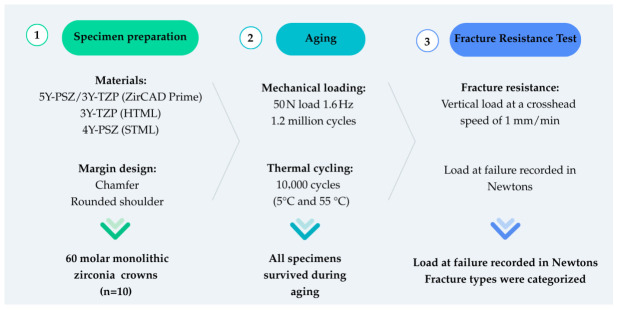
The design of the study.

In this in vitro study, the effects of margin design on the fracture resistance of zirconia restorations with different yttria contents were evaluated. The minimum size to be included in each group was calculated to determine fracture resistance. Since a two-way ANOVA test was planned to investigate the effects of different yttria contents (3Y-TZP, 4Y-PSZ, and 3Y-TZP/5Y-PSZ) and margin designs (chamfer and rounded shoulder) on fracture resistance of zirconia restorations, the minimum required sample size was calculated using the G*Power software version 3.1.9.7 (Kiel University, Kiel, Germany). Assuming an effect size of *f* = 0.40, a Type I error rate of 0.05, and a power of ~0.80, the number of specimens per group was determined to be 10. The study design is schematized in Figure 2.

### 2.2. Specimen Preparation

**Figure 3:**
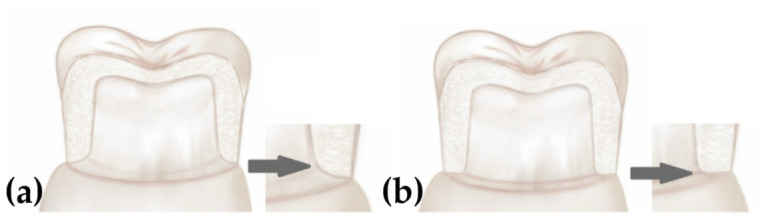
Schematic illustration of the margin preparation designs: (**a**) chamfer and (**b**) rounded shoulder.

In the typodont model (Frasaco, Tettnang, Germany), two right mandibular first molars were prepared according to their anatomical form. The preparation geometry was standardized to include 1 mm occlusal and axial reductions, a total convergence angle of approximately 6°, and rounded internal angles. Two different margin designs, a chamfer and a rounded shoulder, were prepared using 1.0 mm diameter diamond burs (Figure 3).

Following the tooth preparations, study models were fabricated using Type III dental stone (Cerestone, İstanbul, Türkiye). The gypsum casts, along with the corresponding opposing arch model, were then mounted on a semi-adjustable articulator (Stratos 200, Ivoclar Vivadent, Schaan, Liechtenstein). The prepared teeth and models were scanned using a lab scanner (Dental Wings 7 Series, Straumann, Basel, Switzerland) to obtain virtual models. Crown designs were created using dental CAD software (ExoCAD DentalDB 2.4, Plovdiv, Darmstadt, Germany) with standardized parameters: 1 mm occlusal thickness, 1 mm axial wall thickness, and a cement space of 50 μm, according to two different margin designs. The crowns were milled using a milling device (Redon Hybrid, Redon Group, İstanbul, Türkiye) from three different monolithic zirconia discs with varying yttria contents (Table 1).

**Figure 4:**
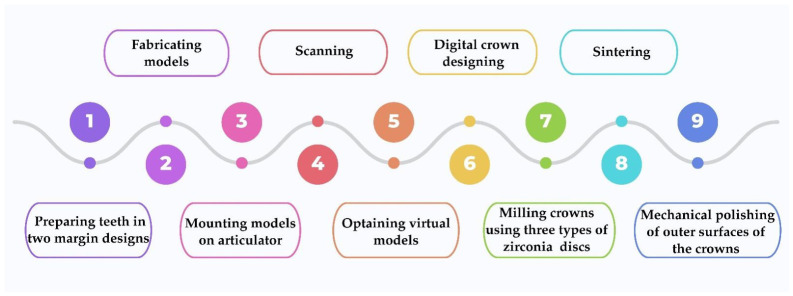
Steps of the zirconia crown fabrication.

Six experimental groups (*n* = 10) were generated according to zirconia type and margin design. Sinterization of the zirconia crowns was performed in a calibrated dental ceramic furnace (Protherm Furnaces, Ankara, Türkiye), according to the manufacturer’s recommended sintering protocols (Table 2). The outer surfaces of the zirconia specimens were mechanically polished. The steps in zirconia crown production are shown in Figure 4.

For the cementation of the zirconia crowns, the scanned typodont teeth were duplicated; a total of 60 resin dies (30 for each margin design) were fabricated using a 3D printer (Formlabs Inc., Somerville, MA, USA) and a model resin (Precision Model Resin, Formlabs Inc., Somerville, MA, USA). Adhesive cementation of the crowns to the corresponding dies was performed following air-particle abrasion, primer application, and adhesive resin cementation. The inner surface of each crown was treated with air-particle abrasion using 50 μm aluminum oxide particles at 2 bars pressure for 15 s from a 10 mm distance using a laboratory sandblasting unit (Basic Mobil, Renfert GmbH, Hilzingen, Germany). Before the primer application, the specimens were ultrasonically cleaned for 10 min in distilled water. After surface pretreatment, a 10-MDP-containing silane coupling agent (Clearfil™ Ceramic Primer Plus, Kuraray Noritake Dental Inc., Tokyo, Japan) was applied to the zirconia surfaces. A 10-MDP-containing tooth primer (Panavia™ V5 Tooth Primer, Kuraray Noritake Dental Inc., Tokyo, Japan) was applied to the 3D-printed resin dies. As the final step of the cementation procedure, the crowns were cemented onto the 3D-printed resin dies using a dual-cure resin cement (Panavia™ V5, Kuraray Noritake Dental Inc., Okayama, Japan). Specimens were light-cured for 10 s from two directions, followed by 10 min of finger pressure to ensure complete chemical polymerization. Each specimen was embedded in a specimen holder filled with autopolymerizing acrylic resin (Integra, Birleşik Grup Dental, Ankara, Türkiye) to ensure stable positioning during aging and mechanical testing.

### 2.3. Aging and Fracture Resistance Test

**Figure 5:**
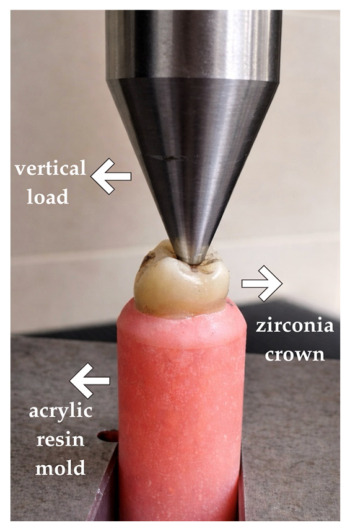
Loading during the fracture resistance test.

Following this preparation, the crowns were subjected to thermal and mechanical artificial aging. Thermal aging was performed using a thermocycler (SD Mechatronik, Feldkirchen-Westerham, Germany) for 10,000 cycles between water baths maintained at 5 °C and 55 °C. Mechanical aging was subsequently conducted in a mastication simulator (Esetron Robotechnologies, Ankara, Turkey) under a load of 50 N at 1.6 Hz, with vertical (4 mm) and horizontal (0.3 mm) movements for 1.2 million cycles. After aging, a vertical load was applied to the central fossa using a 4 mm stainless steel sphere at a crosshead speed of 1 mm/min, and fracture resistance values (maximum loads at failure) were recorded in Newtons using a universal testing machine (Lloyd Instruments, Fareham, UK) (Figure 5).

**Figure 6:**
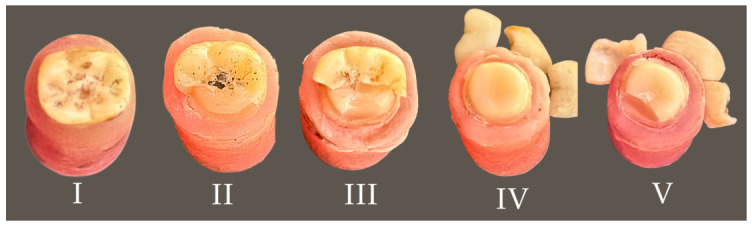
Fracture types of specimens.

Subsequently, the fracture types were assessed by visual inspection (Figure 6) and classified as (I) chipping, (II) crown fracture without debonding, (III) combined crown and die fracture without debonding, (IV) crown fracture with debonding, and (V) combined crown and die fracture with debonding.

Based on the visual classification of fracture types, representative specimens were selected for scanning electron microscopy (SEM) analysis. One specimen with a crown fracture and debonding (Type IV) from each experimental group, along with a single specimen showing chipping (Type I), was included. After gold sputter-coating, the fracture surfaces were examined using a scanning electron microscope (SEM; JSM-6400, JEOL Ltd., Tokyo, Japan) at a magnification of ×1500 to evaluate fracture surface characteristics and crack propagation patterns.

### 2.4. Statistical Analysis

Statistical analyses were performed using SPSS software (IBM SPSS Statistics, Version 20; IBM Corp., Armonk, NY, USA). The normality of the data distribution was confirmed by the Shapiro–Wilk test. Homogeneity of variances was verified with the Levene test (*p* = 0.428). Fracture resistance was analyzed as the dependent variable, considering yttria content and margin design as independent factors. Two-way analysis of variance (ANOVA) was conducted to evaluate the main effects of yttria content and margin design, as well as their interaction effect on fracture resistance. Bonferroni adjustment was used for multiple comparisons. Statistical significance was set at α = 0.05.

## 3. Results

All specimens survived thermal and mechanical aging and were subjected to a fracture resistance test. Two-way ANOVA results of the fracture resistance data showed that there was an interaction between the yttria content and margin design factors (*p* = 0.005, *p* < 0.05) (Table 3).

Descriptive and comparative statistics of the fracture resistance (N) data are presented in Table 4. In the chamfer margin design, the ZP and HTML groups exhibited significantly higher mean fracture resistance values than the STML group (*p* < 0.05). In contrast, no statistically significant difference was observed among the yttria content groups in the rounded shoulder margin design (*p* > 0.05). However, the fracture resistance values of the ZP and HTML groups decreased with the rounded shoulder margin design, and this reduction was statistically significant in the ZP group (*p* < 0.05). In contrast, the STML group showed increased fracture resistance with the rounded shoulder margin design; however, this increase was not statistically significant (*p* > 0.05).

Fracture types of each experimental group are shown in Table 5. One specimen in the ZP-RS group showed chipping. Most of the specimens demonstrated fracture type IV (crown fracture with debonding).

**Figure 7:**
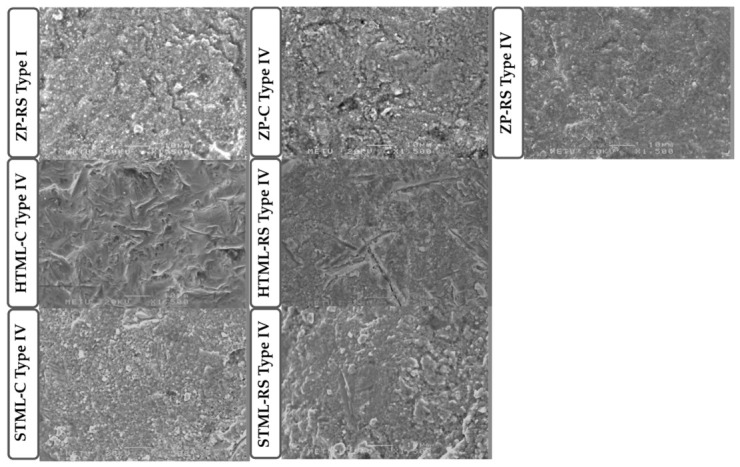
SEM images of the fracture lines.

SEM analysis revealed predominantly brittle fracture features (Figure 7). The HTML specimens showed irregular fracture surfaces with clear crack paths and sharp angular features, and in some areas, the fracture appeared more localized. Compared with this, the ZP group showed a more continuous fracture surface, although changes in crack direction and local texture variations remained evident. The STML specimens displayed a rougher and less uniform morphology. In specimens with Type IV fractures, the surfaces were markedly irregular and disrupted, suggesting rapid fracture progression. The Type I specimen showed a relatively more localized fracture pattern.

## 4. Discussion

The effects of the three different yttria contents (3Y-TZP/5Y-PSZ, 3Y-TZP, and 4Y-PSZ) and two different margin designs (chamfer and rounded shoulder) on the fracture resistance of monolithic zirconia crowns were tested in the present study. Two-way ANOVA showed an interaction between yttria content and margin design factors (*p* < 0.05). Therefore, the null hypothesis was rejected because fracture resistance was affected by the interaction between yttria content and margin design.

The yttria content of zirconia crowns directly affects their translucency and mechanical strength. Zirconia ceramics with higher yttria content exhibit greater translucency, resulting in a higher cubic phase ratio and thereby enhancing esthetic outcomes. However, a higher cubic phase is generally associated with reduced mechanical properties. In contrast, zirconia with lower yttria content exhibits higher mechanical durability but lower translucency. Therefore, the yttria content of zirconia crowns represents a balance between esthetic requirements and mechanical reliability and should be selected based on the clinical indication [23,24]. Especially in the posterior regions, masticatory forces can reach approximately 900 N [18]. 3Y-TZP, 4Y-PSZ, and 3Y-TZP/5Y-PSZ ceramics are indicated for the posterior crowns [4,25]. Thus, in the present study, crowns were fabricated from these zirconia ceramics for mandibular first molars, and fracture resistance was compared to evaluate their performance under posterior loading conditions. In vitro studies should closely replicate intraoral conditions to obtain clinically relevant data. Restorations in the oral environment are continuously exposed to thermal differences, moisture, and repetitive masticatory forces, which may lead to material fatigue, microcrack propagation, and stress accumulation at the tooth–cement–restoration interface over time [11]. Therefore, fracture resistance tests performed without artificial aging may not accurately reflect long-term clinical performance. It is generally accepted that approximately 200,000 to 300,000 chewing cycles correspond to 1 year of intra-oral function [26]. In the present study, thermal and mechanical aging were applied, consisting of 10,000 thermal cycles (5 –55 °C) and 1.2 million mechanical cycles (50 N, 1.6 Hz), corresponding approximately to 5 years of clinical service [18,27]. In the present in vitro study design, the experimental protocol incorporated 3D-printed resin die materials with elasticity comparable to dentin, cyclic loading to simulate functional mastication, and thermocycling to test the wet conditions, thereby enhancing the physiological relevance of the biomechanical simulation and more accurately approximating intraoral conditions.

In previous studies, the mechanical properties of zirconia ceramics with varying yttria ratios have been investigated. These studies mainly focused on evaluating how variations in yttria content affect mechanical characteristics, including fracture resistance [2,3,11,25], fatigue resistance [12,17,18], flexural strength [24,28], fracture toughness, and hardness [4,29]. Badr et al. [11] investigated the fracture resistance of multilayer (5Y-PSZ/3Y-TZP and 5Y-PSZ/4Y-PSZ) and monolithic (4Y-PSZ and 3Y-TZP) zirconia crowns, either thermocycled alone or thermomechanically aged. Material type significantly affected the fracture load. 3Y-TZP exhibited the highest fracture load, followed by monolithic 4Y-PSZ, whereas both multilayer ceramics demonstrated comparatively lower values. The values were not significantly different between the two multilayer ceramics. Furthermore, thermomechanical aging did not significantly influence fracture load within the tested groups. In contrast, in the present study, 3Y-TZP and 3Y-TZP/5Y-PSZ multilayer zirconia ceramics demonstrated comparable fracture resistance, while 4Y-PSZ exhibited lower values. This difference can be attributed to variations in CAM positioning. Badr et al. [11] reported that the multilayer crowns exhibited an occlusal thickness of 3 mm, resulting in the occlusal region being predominantly composed of the mechanically weaker 5Y-PSZ phase, which may have influenced the observed mechanical performance. Spitznagel et al. [18] investigated the fatigue behavior and failure load of multilayer zirconia (4Y-PSZ/5Y-PSZ) crowns at different ceramic thicknesses. They reported that crowns with 1.0 mm and 1.5 mm thickness performed reliably. In comparison, they recommended maintaining a minimum thickness of 1.0 mm for adequate mechanical performance. In the present study, all crowns were fabricated with a standardized thickness of 1.0 mm. Although the manufacturers recommend different minimum thicknesses for posterior crowns depending on the material (0.5 mm for HTML, 1 mm for IPS e.max ZirCAD Prime, and 1.0 mm for STML), a uniform thickness was selected to ensure methodological standardization across the experimental groups. Abad-Coronel et al. [25] also evaluated the fracture resistance of translucent monolithic zirconia crowns containing 3 mol% and 4 mol% yttria. Crowns were designed with a chamfer margin configuration. The mean fracture resistance values were reported as approximately 1745 N and 2387 N for the 3 mol% and 4 mol% yttria-containing zirconia ceramics, respectively. Although both ceramics demonstrated fracture resistance values exceeding the maximum physiological masticatory forces (441–981 N in the molar region) and were considered clinically acceptable, the findings did not align with the general assumption in the literature that increased yttria content reduces fracture resistance. The authors suggested that fracture behavior could not be attributed only to yttria concentration, and that microstructural characteristics, the absence of cementation, and differences in manufacturing and sintering processes play a more significant role in determining mechanical performance. Kongkiatkamon et al. [30] investigated the fracture loads of translucent zirconia crowns from different manufacturers, two 3Y-TZP (Cercon HT and Amann Girbach) and two 5Y-PSZ materials (Cercon XT and Vita YZ XT). Molar crowns with a 1.5 mm thickness and a deep chamfer finish line were subjected to static fracture testing without aging. Fracture load values ranged from approximately 2900 N to 4800 N. Consistent with the findings of Abad-Coronel et al. [25], it was also emphasized that fracture load was a multifactorial outcome and could not be explained solely by yttria content. However, in the present study, despite evaluating zirconia ceramics with comparable yttria contents from different manufacturers, the 3Y-TZP group (HTML) exhibited higher fracture resistance values than the 4Y-PSZ group (STML). This apparent discrepancy further supported the conclusion that fracture resistance was influenced not only by yttria content but also by brand-specific microstructural features and processing parameters. Abdulmajeed et al. [10] investigated how variations in yttria content and material thickness influence the biaxial fracture load of zirconia, both in the presence and absence of simulated mastication. The results demonstrated that both yttria content and thickness significantly influenced fracture load, with lower yttria concentrations and greater thickness resulting in higher mean biaxial fracture loads. 3Y-TZP demonstrated the greatest fracture load, and 4Y-PSZ and 5Y-PSZ showed lower values. Specimens with a thickness of 1.2 mm exhibited significantly higher fracture loads compared to those with a thickness of 0.7 mm across all conditions. During mastication simulation, a substantial proportion of thin specimens fractured, and at a thickness of 0.7 mm, only 3Y-TZP survived the simulated aging. Although mastication simulation did not significantly affect the fracture load of the surviving specimens, the findings indicated that a minimum thickness of 1.2 mm is required for 4Y-PSZ and 5Y-PSZ to ensure adequate mechanical performance. Koo et al. [24] evaluated the effect of long-term hydrothermal aging on conventional and multilayer translucent zirconia ceramics. They demonstrated that specimens derived from the 3Y-TZP/5Y-PSZ transition zone of IPS e.max ZirCAD Prime exhibited biaxial flexural strength, Vickers hardness, and phase transformation behavior comparable to conventional 3Y-TZP (IPS e.max ZirCAD LT) before and after aging. The findings suggested that the 3Y/5Y strength-gradient structure may preserve mechanical properties similar to those of 3Y-TZP. This observation was consistent with the present study, which reported that fracture resistance values of ZP (3Y-TZP/5Y-PSZ) and HTML (3Y-TZP) were not significantly different.

In addition to material composition, the mechanical success of zirconia restorations is also significantly influenced by their design. Restoration design determines how occlusal loads are distributed through factors such as thickness distribution, connector dimensions, and stress concentration areas. These design parameters are directly shaped by the geometry of the tooth preparation, making the preparation configuration one of the crucial factors in the mechanical performance and clinical success of zirconia restorations [8,19,28]. Joy et al. [22] investigated the effect of four different margin designs (flat-ended shoulder, round-ended shoulder, radial shoulder, and deep chamfer) on the fracture resistance of monolithic zirconia crowns. The deep chamfer design yielded the highest fracture resistance values. Salama et al. [21] compared featheredge and radial shoulder margin designs using two 3Y-TZP monolithic zirconia materials, pre-shaded (BruxZir Shaded 16 PLUS) and multilayer (Katana Zirconia HTML PLUS). It was reported that crowns fabricated with a featheredge design exhibited significantly lower marginal gaps and higher fracture resistance after thermocycling. Moreover, no statistically significant difference was observed between the featheredge subgroups of BruxZir and HTML, whereas significant differences were detected between the corresponding radial shoulder subgroups in fracture resistance. This discrepancy indicates that margin designs alone may not be the primary determinant of fracture resistance. Instead, the mechanical outcome depends on the interaction between preparation geometry and material characteristics. A comparable material-dependent variation was reflected in the present results, as chamfer and rounded shoulder preparations yielded similar fracture resistance values in the HTML and STML groups but significantly different outcomes in the ZP group. Gavara et al. [20] evaluated the fracture resistance of no finish line, heavy chamfer, and shoulder marginal designs using 0.5 mm thick zirconia copings fabricated from translucent 3Y-TZP monolithic zirconia discs. Although the chamfer margin design demonstrated the highest fracture resistance, in agreement with the present study, the lower values may be explained by the evaluation of 0.5 mm thick zirconia copings rather than full anatomic contour restorations. Anatomically shaped crowns produced at clinically recommended thicknesses have been reported to provide significantly greater fracture resistance [22,25,30,31]. AbdElaziz et al. [31] investigated gradient multilayer zirconia (Katana YML, Kuraray Noritake) crowns fabricated with margin designs (rounded shoulder, chamfer, and knife-edge) and cement space thicknesses. Following thermocycling and subsequent chewing simulation (118,000 cycles), the margin design significantly influenced fracture resistance, with rounded shoulder preparations exhibiting the highest mean fracture load, followed by chamfer and knife-edge designs. The present findings agree with those of AbdElaziz et al. [31] and confirm that finish line design significantly affects fracture resistance. The present study also revealed that the influence of marginal design depends on the yttria content of zirconia materials. However, no significant difference in margin design was observed between the HTML and STML groups, whereas chamfer preparation showed significantly higher fracture resistance in the ZP groups. The finding suggested that the influence of margin design on fracture resistance may be material-dependent.

Fracture types were also evaluated in the present study. Beuer et al. [32] noted that occlusal loading resulted in stress accumulation at the contact area, with crack initiation at the occlusal surface and subsequent propagation through the restoration. In the present study, fracture lines also originated from the occlusal loading area. Type IV failure (crown fracture with debonding) was predominantly observed in the HTML and STML restorations, whereas a more heterogeneous distribution of fracture types was detected in the ZP restorations. Variations in yttria content and the resulting phase composition of zirconia may account for the differences in fracture behavior. In the ZP group (5Y-PSZ/3Y-TZP), in which the occlusal surface was composed of 5Y-PSZ, chipping (Type I) was observed in one specimen. In strength-gradient zirconia systems, the presence of a 5Y-PSZ occlusal layer influences crack initiation and propagation, which may explain the greater variability detected in the ZP restorations [33]. Such crack progression may facilitate stress transfer toward the adhesive interface, leading to debonding-associated failures. As yttria content increases, the material becomes more brittle, and cracks propagate more readily, leading to a greater number of fragments after fracture. In contrast, when the tetragonal phase is more predominant, crack propagation is limited, resulting in fewer fracture lines and fewer fragments [11]. Alzhairi et al. [34] stated that lower yttria content zirconia exhibited more stable fracture behavior with controlled veneer-related failures, while higher yttria content promoted brittle bulk fractures attributed to translucency-related phase composition changes. The findings were consistent with the present study, in which increasing yttria content was similarly associated with a shift toward more brittle fracture patterns and a higher prevalence of catastrophic failures. The results of the present study indicated that higher yttria content was associated with more brittle fracture behavior. Although HTML (3Y-TZP) was generally associated with higher fracture toughness and more stable crack propagation, the greater number of fragments observed in the HTML group suggested that fracture behavior cannot be attributed only to yttria content.

The present study has some limitations. 3D-printed resin dies were used instead of natural teeth; however, they do not fully replicate the biomechanical behavior of natural dentin or simulate the periodontal ligament. Fracture resistance is an essential parameter for evaluating the mechanical durability of zirconia ceramics. Still, additional tests, such as fracture toughness and flexural strength, are needed to characterize their mechanical properties fully. To generalize the results to all zirconia materials, further investigations are required on zirconia materials from other manufacturers and brands with different yttria contents.

## 5. Conclusions

Based on the findings of the present study, the following conclusions were drawn.

(1) Yttria content affected fracture resistance, and the effect of margin design varied depending on the zirconia type.
(2) All experimental groups survived thermal and mechanical aging simulating 5 years of clinical service, and fracture resistance values indicated that all the tested zirconia crowns can withstand masticatory forces when used in posterior regions.
(3) 3Y-TZP-containing materials (ZP and HTML) showed significantly higher fracture resistance values for the chamfer-margin design. However, the rounded shoulder margin design resulted in higher fracture resistance values for the tested 4Y-PSZ (STML) ceramic.

## Figures and Tables

**Table 1:** Properties of the zirconia discs used.

Brand/ Manufacturer/Abbreviation	Content	Composition	Lot Number
IPS e.max ZirCAD Prime Ivoclar Vivadent, Schaan, Liechtenstein ZP	Occlusal: 5Y-PSZ Dentin: 3Y-TZP	Zirconium oxide (ZrO_2_): 86.0–93.5%, Yttrium oxide (Y_2_O_3_): >6.5–≤8.3%, Hafnium oxide (HfO_2_): ≤5.0%, Aluminum oxide (Al_2_O_3_): ≤1.0%, Other oxides: ≤1.0%	Z061CN
Katana HTML PLUS Kuraray Noritake Dental, Inc., Tokyo, Japan HTML	3Y-TZP	Zirconium oxide (ZrO_2_): 87–89%, Yttrium oxide (Y_2_O_3_): 5.2%, Hafnium oxide (HfO_2_): <2%, Other oxides (e.g., aluminum oxide): ≤2%	EMYJI
Katana STML Kuraray Noritake Dental, Inc., Tokyo, Japan STML	4Y-PSZ	Zirconium oxide (ZrO_2_) + Hafnium oxide (HfO_2_): 88–93%, Yttrium oxide (Y_2_O_3_): 7–10%, Other oxides: ≤2%	EMBUD

**Table 2:** Sintering parameters of the zirconia crowns used.

Materials	Phase	Heating Rate (°C/min)	Sintering Temperature (°C)	Holding Time (min)	Cooling Rate (°C/min)	Total Time
ZP	1	10	900	30	10	~9 h 50 min
	2	3.3	1500	120	8.3	
HTML		10	1500	120	10	~7 h
STML		10	1550	120	10	~7 h

**Table 3:** Two-way ANOVA results for the fracture resistance data.

Source	Type III Sum of Squares	df	Mean Square	F	Sig.	Partial Eta Squared
Corrected Model	3,877,720.933 ^a^	5	775,544.187	4.466	0.002	0.293
Intercept	206,142,563.267	1	206,142,563.267	1187.071	<0.001	0.956
Yttria content	1,687,847.233	2	843,923.617	4.860	0.011	0.153
Margin design	173,666.400	1	173,666.400	1.000	0.322	0.018
Yttria content × Margin design	2,016,207.300	2	1,008,103.650	5.805	0.005	0.177
Error	9,377,447.800	54	173,656.441			
Total	219,397,732.000	60				
Corrected Total	13,255,168.733	59				

^a^ R Squared = 0.293 (Adjusted R Squared = 0.227).

**Table 4:** Descriptive and comparative statistics of the fracture resistance (N) data (*n* = 10).

Yttria Content	Margin Design	
	Chamfer (C)	Rounded Shoulder (RS)
ZP (3Y-TZP/5Y-PSZ)	2208.5 (±501.9) A, a	1662.8 (±293.8) A, b
HTML (3Y-TZP)	2069.6 (±463.3) A, a	1940.9 (±341.6) A, a
STML (4Y-PSZ)	1444 (±303.2) B, a	1795.6 (±529.6) A, a

The same uppercase letters indicate that fracture resistance values were not significantly different between the yttria content groups in the same margin design group (*p* > 0.05). The *p* values for chamfer margin design experimental groups: HTML-STML, *p* = 0.004; HTML-ZP, *p* = 1; STML-ZP, *p* < 0.001; for rounded shoulder margin design: HTML-STML, *p* = 1, HTML-ZP, *p* = 0.424, STML-ZP, *p* = 1. The same lowercase letters indicate that the fracture resistance values were not significantly different among the margin design groups in the same yttria content group (*p* > 0.05). The *p* values between margin designs within each yttria content group: HTML (Chamfer-Rounded shoulder), *p* = 0.493; STML (Chamfer-Rounded shoulder), *p* = 0.065; ZP (Chamfer-Rounded shoulder), *p* = 0.005.

**Table 5:** Distribution of fracture types among the experimental groups.

Groups	I	II	III	IV	V
ZP-C	0	3	0	5	2
ZP-RS	1	3	2	2	2
HTML-C	0	2	2	4	2
HTML-RS	0	2	2	4	2
STML-C	0	4	0	6	0
STML-RS	0	0	0	7	3

## Data Availability

The original contributions presented in the study are included in the article, further inquiries can be directed to the corresponding author.

## Abbreviations

The following abbreviations are used in this manuscript:

10-MDP	10-Methacryloyloxydecyl dihydrogen phosphate
3D	Three-dimensional
ANOVA	Analysis of variance
C	Chamfer
CAD-CAM	Computer-aided design-Computer-aided manufacturing
HTML	High translucency multilayered
N	Newton
RS	Rounded shoulder
STML	Super translucency multilayered
Y-PSZ	Yttria-partially stabilized zirconia
Y-TZP	Yttria-stabilized tetragonal zirconia polycrystals
ZP	IPS e.max ZirCAD Prime
